# A National Ambulatory Surgery Sample Cost Analysis of Outpatient Facial Feminization Surgery

**DOI:** 10.7759/cureus.82849

**Published:** 2025-04-23

**Authors:** Sandhya Kalavacherla, Justin M Camacho, Justin Cordero, Sruthi Kalavacherla, Lucy Sheahan, Amanda Gosman

**Affiliations:** 1 Department of Otolaryngology, Head and Neck Surgery, University of California San Diego School of Medicine, San Diego, USA; 2 Department of Medicine, Drexel University College of Medicine, Philadelphia, USA; 3 Department of Surgery, University of California Riverside School of Medicine, Riverside, USA; 4 Department of Biology, Massachusetts Institute of Technology, Cambridge, USA; 5 Department of Plastic and Reconstructive Surgery, University of California San Diego School of Medicine, San Diego, USA

**Keywords:** cost analysis, facial feminization surgery, gender-affirming care, insurance reimbursement, transgender

## Abstract

Introduction: Facial feminization surgery (FFS) alleviates gender dysphoria, but insurance coverage is minimal and underreported. This study analyzes total charges and primary payer distributions for outpatient FFS care using a national database.

Materials and methods: Data from the 2017-2018 National Ambulatory Surgery Sample were analyzed to identify transgender patients undergoing FFS using ICD-10 and CPT codes. Demographics, surgery center location, and total charges were stratified by procedure and primary payer type and compared using descriptive statistics.

Results: A total of 3,359 encounters were identified with a median patient age of 42 years and a median (interquartile range) charge of $24,679 ($15,716, $39,442). Private insurance was the most common payer (N=1657, 50%), followed by self-payers (N=667, 20%), Medicare (N=540, 16%), and Medicaid (N=286, 8.3%). Median costs were highest for self-payers ($27,736 ($17,392, $39,385)), followed by private insurance ($26,989 ($17,798, $44,933)) and Medicaid ($26,968 ($16,756, $46,467)). Medicare had the lowest median charge ($17,467 ($10,322, $29,210)). Charges differed significantly by income (p<0.001) and location (p<0.001), with higher earners and central metropolitan areas incurring the highest costs ($25,249 and $26,782, respectively). Rhinoplasty (N=1990, 59%) and brow lifts (N=363, 11%) were the most common procedures. Brow lifts had the lowest median cost ($11,834 ($8,366, $18,317)) and were most often covered by Medicare (N=217, 60%). The total charges for rhinoplasty were $23,050, with private insurance as the primary payer (N=1017, 51%).

Conclusions: This study is the first to characterize total charges and payer distributions for outpatient FFS. This broad analysis highlights the significant financial burden of FFS, especially on self-paying patients.

## Introduction

Facial feminization surgery (FFS) refers to a collection of craniomaxillofacial procedures that are designed to alleviate the internal incongruencies many transfeminine patients face as a result of gender dysphoria. The utilization of surgical procedures for facial feminization was pioneered and optimized in the early 1980s by Dr. Douglas Ousterhout [1]. Despite its relatively sparse history in early literature, recent research suggests an increasing demand for gender-affirming facial procedures [2,3].

It is well-documented that the field of gender-affirming surgery has been pivotal in alleviating the psychosocial turmoil inflicted by gender dysphoria in transgender patients [4-6]. A patient’s face is an essential component of identity expression and how they present themselves to the world [7]. Despite this, insurance coverage of FFS remains limited, while insurance coverage for top and bottom surgery has improved over the years. This is primarily attributed to the belief that this collection of procedures is cosmetic rather than a medical necessity [7,8]. Furthermore, navigating the insurance approval process requires considerable time and effort from both the patient and the provider, and coverage is subject to the complexities of state and federal laws. These barriers to access to these surgeries present a disconcerting threat to the mental health and social well-being of transfeminine patients.

Furthermore, Hu et al. highlight that the average cost burden of gender-affirming surgery to the patient has risen from $13,657 in 2008 to $50,789 in 2017, which is a nearly fourfold increase [9]. In gender-affirming surgery research, there is a paucity of literature devoted to understanding the cost burden, specifically of FFS. Additionally, many studies that have performed cost analysis for varying gender-affirming procedures have been limited to assessing inpatient rather than outpatient costs. Therefore, this study aims to elucidate the total cost and primary payer distributions for outpatient FFS, helping the medical community understand the financial implications associated with these highly sought-after and life-altering medical procedures.

## Materials and methods

The National Ambulatory Surgery Sample (NASS) is part of the Healthcare Cost and Utilization Project (HCUP) and provides annual, national estimates of ambulatory surgery encounters performed in hospital-owned facilities. NASS data from 2017-2018 were first queried for transgender patients using International Classification of Diseases, 10th Revision (ICD-10) codes. ICD-10 codes F64 and Q56.3 refer more explicitly to gender dysphoria but were not available within the NASS 2016-2018 databases [10,11]. The choice was then made to query for code Z79.899, which refers to patients undergoing any long-term (current) drug therapy, which has been reported as a medical coding alternative to F64 for transgender patients [11]. Among encounters coded with the ICD-10 code Z79.899, a group of Current Procedural Terminology (CPT) codes related to FFS was queried (Table 1).

**Table 1:** CPT codes related to FFS queried from the NASS database CPT: Current Procedural Terminology, FFS: facial feminization surgery, NASS: National Ambulatory Surgery Sample

CPT code	Procedure description
15821	Blepharoplasty, lower eyelid; with extensive herniated fat pad
15822	Blepharoplasty, upper eyelid
15823	Blepharoplasty, upper eyelid; with excessive skin weighting down lid
15824	Rhytidectomy; forehead
15825	Rhytidectomy; neck with platysmal tightening
15826	Rhytidectomy; glabellar frown lines
15828	Rhytidectomy; cheek, chin, and neck
15876	Suction assisted lipectomy; head and neck
21083	Impression and custom preparation; palatal lift prosthesis
21087	Impression and custom preparation; obturator prosthesis
21120	Genioplasty; augmentation (autograft, allograft, prosthetic material)
21121	Genioplasty; sliding osteotomy, single piece
21122	Genioplasty; sliding osteotomies, two or more pieces (e.g., wedge excision or bone wedge reversal for asymmetrical chin)
21123	Genioplasty; sliding, augmentation with interpositional bone grafts (includes obtaining autografts)
21125	Augmentation, mandibular body or angle; prosthetic material
21127	Augmentation, mandibular body or angle; with bone graft, onlay or interpositional (includes obtaining autograft)
21137	Reduction forehead; contouring only
21138	Reduction forehead; contouring and application of prosthetic material or bone graft (includes obtaining autograft)
21139	Reduction forehead; contouring and setback of anterior frontal sinus wall
21141	Reconstruction midface, LeFort I; single piece, segment movement in any direction (e.g., for long face syndrome), without bone graft
21142	Reconstruction midface, LeFort I; two pieces, segment movement in any direction, without bone graft
21143	Reconstruction midface, LeFort I; three or more pieces, segment movement in any direction, without bone graft
21145	Reconstruction midface, LeFort I; single piece, segment movement in any direction, requiring bone grafts (includes obtaining autografts)
21146	Reconstruction midface, LeFort I; two pieces, segment movement in any direction, requiring bone grafts (includes obtaining autografts)
21147	Reconstruction midface, LeFort I; three or more pieces, segment movement in any direction, requiring bone grafts (includes obtaining autografts)
21150	Reconstruction midface, LeFort II; anterior intrusion (e.g., Treacher Collins syndrome)
21151	Reconstruction midface, LeFort II; any direction, requiring bone grafts (includes obtaining autografts)
21154	Reconstruction midface, LeFort III; without bone graft
21155	Reconstruction midface, LeFort III; requiring bone grafts (includes obtaining autografts)
21159	Reconstruction midface, LeFort III; with forehead advancement (e.g., monobloc)
21160	Augmentation malar (cheek) area; prosthetic material
21172	Reconstruction superior-lateral orbital rim and lower forehead; with bone graft (includes obtaining autograft)
21175	Reconstruction, bifrontal, superior-lateral orbital rims and lower forehead; with osteotomies and bone grafts (includes obtaining autografts)
21179	Reconstruction of mandibular rami, horizontal, vertical, C, or L osteotomy; with bone graft (includes obtaining autograft)
21180	Reconstruction of mandibular rami and/or body, sagittal split; with internal rigid fixation
21188	Reconstruction of mandibular condyle (e.g., for ankylosis)
21208	Osteoplasty, facial bones; augmentation (autograft, allograft, or prosthetic implant)
21209	Osteoplasty, facial bones; reduction

These CPT codes encompass procedures performed as part of the most commonly used FFS, including rhinoplasty, midface reconstruction, lipectomy, brow lift, mandibular reconstruction, genioplasty, osteoplasty, forehead reconstruction, and malar augmentation. Using these broad category ICD and CPT codes in theory allowed us to capture transgender females undergoing long-term hormone therapy in addition to FFS.

Only encounters coded with a single CPT code were included in the study to determine the total cost per code. Encounters were then grouped based on the role of the CPT code in each of the 11 procedures above. From the queried sample, we collected demographic data on patient age and average income by zip code, which were stratified into the following categories: $1-$42,999, $43,000-$53,999, $54,000-$70,999, and $71,000 or more. Additionally, we collected data on the region where the ambulatory surgery was performed for each encounter, which NASS classified into large central metropolitan, large fringe metropolitan, medium metropolitan, micropolitan, non-metropolitan, or small metropolitan. The primary payer for each encounter, including Medicare, Medicaid, self-pay, private insurance, or other, as well as the total charge in U.S. dollars, was identified.

All data processing and statistical analyses were performed using R statistical software version 4.1.3 (R Foundation for Statistical Computing, Vienna, Austria, https://www.R-project.org/). The demographic data, ambulatory surgery center location, primary payer data, and total charges for each encounter were stratified by procedure type and were compared across procedure types using descriptive statistics. Similarly, demographic data, total charges, and ambulatory surgery center location were stratified by primary payer types and compared between primary payer types. Analysis of variance tests were used to compare whether total charges differed between groups, taking into account age, average income based on the patient's zip code of residence, location of the ambulatory surgery center, and primary payer type. Significance was defined as p<0.05.

## Results

A total of 3,359 relevant procedure encounters were identified (Table 2).

**Table 2:** Overall demographic characteristics, total charges, primary payers, and procedure types across all identified encounters ^1^ n (%); median (IQR) IQR: interquartile range

Characteristic	N=3,359^1^
Age (year)	42 (26, 58)
Average income by zip code	-
$1-$42,999	576 (17%)
$43,000-$53,999	727 (22%)
$54,000-$70,999	946 (28%)
$71,000+	1,067 (32%)
Unknown	43 (1.3%)
Location of surgery center	-
Large central metropolitan	950 (28%)
Large fringe metropolitan	1,003 (30%)
Medium metropolitan	740 (22%)
Micropolitan	261 (7.8%)
Not metropolitan or micropolitan	165 (4.9%)
Small metropolitan	231 (6.9%)
Unknown	9 (0.3%)
Primary payer	-
Medicaid	286 (8.5%)
Medicare	540 (16%)
Private insurance	1,657 (49%)
Self pay	667 (20%)
Other	192 (5.7%)
Unknown	17 (0.5%)
Total charge ($)	24,679 (15,716, 39,442)
Procedure type	-
Brow lift	363 (11%)
Forehead reconstruction	21 (0.6%)
Genioplasty	62 (1.8%)
Lipectomy	296 (8.8%)
Malar reconstruction	8 (0.2%)
Mandibular contouring	77 (2.3%)
Maxillary and mandibular reconstruction	68 (2.0%)
Midface reconstruction	421 (13%)
Osteoplasty	53 (1.6%)
Rhinoplasty	1,990 (59%)

The median (IQR) age of patients was 42 (26, 58) years. The median total charge across all encounters was $24,679 ($15,716, $39,442). Private insurance was the most common primary payer (N=1657, 50%), followed by self-payers (N=667, 20%), Medicare (N=540, 16%), and Medicaid (N=286, 8.3%). Most procedures were performed in large metropolitan areas (N=1953, 58%). Among the nine procedure types studied for facial feminization, rhinoplasty was the most commonly used (N=1990, 59%) in all encounters, followed by brow lifts (N=363, 11%).

The total charge differed significantly by primary payer type (p<0.001). Self-payers had the highest median cost at $27,736 ($17,392, $39,385), followed by private insurance ($26,989 ($17,798, $44,933)) and Medicaid ($26,968 ($16,756, $46,467)) (Table 3).

**Table 3:** Demographic characteristics, total charges, and procedure types stratified by primary payer type across all identified encounters ^1^ median (IQR); n (%), ^2^ Kruskal-Wallis rank sum test; Pearson’s chi-squared test IQR: interquartile range

Characteristic	Medicaid, N=286^1^	Medicare, N=540^1^	Private insurance, N=1,657^1^	Self, N=667^1^	Other, N=192^1^	p-value^2^
Total charge ($)	26,968 (16,756, 46,467)	17,467 (10,322, 29,210)	26,989 (17,798, 44,933)	27,736 (17392, 39385)	25,486 (18,157, 39,413)	<0.001
Age (years)	31 (19, 46)	70 (65, 77)	37 (23, 52)	40 (27, 53)	42 (29, 53)	<0.001
Average income by zip code						<0.001
$1-$42,999	87 (31%)	132 (24%)	236 (14%)	90 (14%)	27 (14%)	
$43,000-$53,999	81 (28%)	138 (26%)	359 (22%)	96 (14%)	50 (27%)	
$54,000-$70,999	85 (30%)	145 (27%)	458 (28%)	188 (28%)	62 (33%)	
$71,000+	32 (11%)	117 (22%)	583 (35%)	286 (43%)	48 (25%)	
Unknown	1 (0.3%)	8 (1.5%)	21 (1.3%)	7 (1%)	5 (1.6%)	
Location of surgery center						<0.001
Large central metropolitan	94 (33%)	108 (20%)	452 (27%)	247 (37%)	47 (25%)	
Large fringe metropolitan	65 (23%)	152 (28%)	486 (29%)	246 (37%)	53 (28%)	
Medium metropolitan	65 (23%)	121 (22%)	414 (25%)	91 (14%)	40 (21%)	
Micropolitan	30 (10%)	56 (10%)	127 (7.7%)	25 (3.8%)	22 (12%)	
Not metropolitan or micropolitan	13 (4.5%)	53 (9.8%)	72 (4.4%)	15 (2.3%)	10 (5.3%)	
Small metropolitan	19 (6.6%)	50 (9.3%)	102 (6.2%)	41 (6.2%)	17 (9.0%)	
Unknown	0	0	4 (0.2%)	2 (0.3%)	3 (1.6%)	
Procedure type						<0.001
Blepharoplasty	81 (22%)	1,759 (74%)	1,019 (36%)	270 (17%)	74 (22%)	
Brow ptosis repair	9 (3.1%)	217 (40%)	98 (5.9%)	22 (3.3%)	17 (8.9%)	
Forehead reconstruction	2 (0.7%)	8 (1.5%)	8 (0.5%)	1 (0.1%)	2 (1.0%)	
Genioplasty	8 (2.8%)	0 (0%)	29 (1.8%)	22 (3.3%)	3 (1.6%)	
Lipectomy	5 (1.7%)	15 (2.8%)	64 (3.9%)	179 (27%)	28 (15%)	
Malar reconstruction	0 (0%)	0 (0%)	3 (0.2%)	4 (0.6%)	1 (0.5%)	
Mandibular contouring	9 (3.1%)	29 (5.4%)	36 (2.2%)	3 (0.4%)	0 (0%)	
Maxillary and mandibular reconstruction	4 (1.4%)	23 (4.3%)	39 (2.4%)	0 (0%)	2 (1.0%)	
Midface reconstruction	57 (20%)	6 (1.1%)	340 (21%)	7 (1.0%)	10 (5.2%)	
Osteoplasty	7 (2.4%)	12 (2.2%)	23 (1.4%)	7 (1.0%)	4 (2.1%)	
Rhinoplasty	185 (65%)	230 (43%)	1,017 (61%)	422 (63%)	125 (65%)	
Rhytidectomy	2 (0.5%)	69 (2.9%)	156 (5.5%)	613 (40%)	68 (20%)	

Medicare had the lowest cost of $17,467 ($10,322, $29,210) and the highest median age of 70 years (65, 77). Self-payers had the highest proportion of patients living in zip codes with average incomes >$71,000 (N=286, 43%), followed by those with private insurance (N=583, 36%). Additionally, self-payers were more likely to be older than those paying with private insurance or Medicaid (40 vs. 37 and 31 years, p<0.001).

Total charges differed significantly by average income, based on patients’ zip codes (p<0.001). Patients residing in zip codes with the highest average income (>$71,000) had the highest mean total charge at $25,249 ($16,399, $38,552). Those residing in zip codes with the lowest average income (<$42,000) had the next highest total charge of $25,489 ($15,807, $38,847). However, total charges differed by the location of the ambulatory center where the procedure was performed (p<0.001), with decreasing costs associated with smaller regions. Large central and fringe metropolitan areas incurred the highest median total charges of $26,782 ($15,981, $44,316) and $24,820 ($15,951, $38,340), respectively, while micropolitan ($22,443 ($15,817, $33,629)) and small metropolitan areas ($23,549 ($15,520, $35,174)) had lower median total charges. Rhinoplasty and brow lift were the most frequently utilized procedures (Table 4).

**Table 4:** Baseline encounter characteristics, total charges, and primary payers stratified by FFS type across all identified encounters ^1 ^n (%); median (IQR), ^2^ Kruskal-Wallis rank sum test FFS: facial feminization surgery, IQR: interquartile range

	Brow lift, N=363^1^	Forehead reconstruction, N=21^1^	Genioplasty, N=62^1^	Lipectomy, N=296^1^	Malar reconstruction, N=8^1^	Mandibular contouring, N=77^1^	Maxillary and mandibular reconstruction, N=68^1^	Midface reconstruction, N=421^1^	Osteoplasty, N=53^1^	Rhinoplasty, N=1,990^1^	p-value^2^
Total charge ($)	11,834 (8,366, 18,317)	37,523 (20,943, 47,063)	30,617 (20,418, 49,489)	25,323 (15,228, 37,497)	21,697 (10,398, 29,111)	48,609 (32,408, 71,509)	30,585 (21,021, 51,115)	52,878 (36,378, 71,069)	36,975 (22,232, 49,647)	23,050 (16,136, 32,960)	<0.001
Primary payer											
Medicaid	9 (2.5%)	2 (9.5%)	8 (13%)	5 (1.7%)	0 (0%)	9 (12%)	4 (5.9%)	57 (14%)	7 (13%)	185 (9.3%)	
Medicare	217 (60%)	8 (38%)	0 (0%)	15 (5.1%)	0 (0%)	29 (38%)	23 (34%)	6 (1.4%)	12 (23%)	230 (12%)	
Private insurance	98 (27%)	8 (38%)	29 (47%)	64 (22%)	3 (38%)	36 (47%)	39 (57%)	340 (81%)	23 (43%)	1,017 (51%)	
Self pay	22 (6.1%)	1 (4.8%)	22 (35%)	179 (61%)	4 (50%)	3 (3.9%)	0 (0%)	7 (1.7%)	7 (13%)	422 (21%)	
Other	17 (4.7%)	2 (9.5%)	3 (4.8%)	28 (9.6%)	1 (13%)	0 (0%)	2 (2.9%)	10 (2.4%)	4 (7.5%)	125 (6.3%)	
Unknown	0	0	0	3 (1%)	0	0	0	0	0	10 (0.5%)	
Age	69 (61, 76)	53 (44, 62)	32 (19, 46)	52 (43, 60)	53 (46, 60)	60 (47, 67)	57 (47, 68)	24 (18, 36)	52 (30, 60)	38 (25, 54)	<0.001
Average income by zip code											<0.001
$1-$42,999	95 (26%)	4 (19%)	12 (19%)	51 (17%)	0 (0%)	16 (21%)	16 (24%)	58 (14%)	15 (28%)	309 (16%)	
$43,000-$53,999	92 (25%)	3 (14%)	12 (19%)	35 (12%)	3 (38%)	19 (25%)	17 (25%)	76 (18%)	9 (17%)	461 (23%)	
$54,000-$70,999	97 (27%)	6 (29%)	16 (26%)	82 (28%)	3 (38%)	20 (26%)	12 (18%)	152 (36%)	13 (25%)	545 (27%)	
$71,000+	72 (20%)	8 (38%)	21 (34%)	124 (42%)	2 (25%)	22 (29%)	20 (29%)	127 (30%)	15 (28%)	656 (33%)	
Unknown	7 (1.9%)	0	1 (1.6%)	4 (1.4%)	0	0	3 (4.4%)	8 (1.9%)	1 (1.9%)	19 (0.95%)	
Location of surgery center											<0.001
Large central metropolitan	75 (21%)	12 (57%)	22 (35%)	108 (37%)	4 (50%)	23 (30%)	25 (37%)	124 (29%)	16 (30%)	541 (27%)	
Large fringe metropolitan	112 (31%)	3 (14%)	19 (31%)	101 (34%)	2 (25%)	24 (31%)	17 (25%)	111 (26%)	11 (21%)	603 (30%)	
Medium metropolitan	71 (20%)	2 (9.5%)	11 (18%)	38 (13%)	0 (0%)	16 (21%)	12 (18%)	125 (30%)	11 (21%)	454 (23%)	
Micropolitan	40 (11%)	2 (9.5%)	3 (4.8%)	12 (4.1%)	0 (0%)	5 (6.5%)	4 (5.9%)	29 (6.9%)	8 (15%)	158 (8.0%)	
Not metropolitan or micropolitan	34 (9.4%)	1 (4.8%)	3 (4.8%)	14 (4.7%)	1 (13%)	2 (2.6%)	5 (7.74%)	14 (3.3%)	3 (5.7%)	88 (4.4%)	
Small metropolitan	31 (8.5%)	1 (4.8%)	4 (6.5%)	22 (7.5%)	1 (13%)	7 (9.1%)	2 (2.9%)	18 (4.3%)	3 (5.7%)	142 (7.2%)	
Unknown	0	0	0	1 (0.3%)	0	0	3 (4.4%)	0	1 (1.9%)	4 (0.20%)	

Brow lifts had the lowest median total charge of $11,834 ($8,366, $18,317) and a high proportion of Medicare payment (N=217, 60%). Rhinoplasty had a median cost of $23,050 ($16,135, $32,960), with the majority of patients paying with private insurance (N=1017, 51%). Procedures related to midface and maxillary/mandibular reconstruction had the highest median total costs of $52,878 ($36,378, $71,069) and $48,609 ($32,408, $71,509), respectively, with large proportions of private insurance primary payers (N=340, 81% and N=39, 57%).

## Discussion

In this retrospective database analysis of the costs of procedures associated with FFS from 2017 to 2018, we report a high average total cost of $24,679. This broad analysis of ICD and CPT codes highlights the theoretical cost burden of FFS for transfeminine patients. Specifically, our findings underscore the greater cost burden among self-payers, who were billed a median of $27,736 (17392, 39385), representing the highest total charge across all primary payer types. Our data further suggests that patients with Medicaid and those residing in zip codes with the lowest average income also experience a higher cost burden for procedures encompassed by FFS, suggesting inequity that may serve as an additional barrier to care. On the other hand, Medicare, which was the third most common primary payer type, had the lowest median total charge of $17,467. To our knowledge, this analysis is the first to report costs and primary payer distributions associated with the commonly performed outpatient FFS.

FFS is one of the most pivotal procedures during the transition period for the transfeminine and gender-diverse patient. It is thus crucial that physicians and other healthcare team members who provide gender-affirming care analyze and anticipate the cost trends associated with insurance coverage for FFS to ensure equitable access to care. This is especially important in a healthcare system where insurance policy information regarding gender-affirming surgery is often convoluted and prohibitively difficult for patients to navigate [12,13]. Our analysis shows that the costs and coverage of these procedures are complicated by factors such as variability in insurance coverage, region, and whether the procedures are performed inpatient or outpatient. Hu et al. identified that only 10% of inpatient gender-affirming surgeries between 2015 and 2017 included FFS, consisting of only 110 procedures, while over 3,300 outpatient procedures were determined by the authors within the NASS database [9]. This analysis of outpatient costs is especially relevant, as a significant proportion of FFS procedures are performed on an outpatient basis in the United States.

A noteworthy feature of our findings was the variation in cost for FFS by the location where the outpatient surgery was performed. Interestingly, surgeries performed in large central and fringe metropolitan areas had higher mean total charges compared to those performed in micropolitan and small metropolitan centers. The reasons underlying the regional cost variation are unclear and likely due to several factors. Regional variation in healthcare costs for other conditions has previously been attributed to a supply and demand model, where increased demand for a certain type of care and the system's limited capacity to provide it result in higher costs [14]. Such a pattern may be reflected in our findings, which could reflect either provider or patient distribution. Forms of government-funded insurance, namely Medicaid and Medicare, were the least common payers for procedures related to FFS in this analysis. Additionally, when analyzing the total costs by primary payer type, Medicaid had one of the highest median total charges of $26,968, which was very close to the total costs for self-payers and private insurance. Given these differential coverages and total charges by primary payer, state policies governing insurance may play a role in this. A previous study by Gadkaree et al. identified a relationship between a lack of coverage for FFS and state laws that limit gender-affirming care [13]. Elucidation of these factors warrants further investigation, particularly given the current political climate, with an increasing number of states enacting legislation related to gender-affirming care [15]. Although not previously reported for outpatient FFS, differences in total costs between Medicare, private insurance, and self-pay have been documented in plastic surgery procedures, such as facial fracture repair, scar revision, and breast surgery [16]. Regarding the costs billed to self-payers and Medicaid compared to those for Medicare and private insurance, there is an opportunity to explore further how healthcare entities view different insurance programs and determine where to focus efforts to reduce healthcare spending.

One limitation of this study is that the NASS data were de-identified and limited to outpatient procedures, with the number of gender-affirming facial procedures extrapolated based on CPT and ICD-10 codes. This data, therefore, is only able to capture a subset of FFS, the accuracy of which relies on the assumption that procedures were coded correctly. Given the many options for alternative coding for transfeminine patients and our use of ICD-10 code Z79.899, which refers to patients undergoing any long-term (current) therapy, we cannot confirm that these patients included in our analysis are, in fact, transfeminine. However, we reasonably assume that encounters coded with Z79.899 and CPT codes related to FFS capture a sizable proportion of transfeminine patients. Additionally, NASS stratifies data by encounter rather than by patient, which limits our ability to discern multiple encounters for a single patient and comprehensively capture the cost for an average transfeminine patient who was treated during different encounters. Additionally, while our data demonstrates the differential total costs billed to each primary payer and the number of encounters associated with each payer type, we cannot determine coverage offered by each primary payer, as NASS does not contain data on whether other payers approved or denied coverage. However, these data provide a useful proxy for analyzing and comparing cost differences, particularly since the NASS dataset remains the most comprehensive resource to date for assessing national trends in outpatient FFS costs.

## Conclusions

We are the first to analyze total costs and payer distributions for outpatient FFS, highlighting the substantial financial burden it poses, particularly for self-paying patients. Overall, these data serve as an important preliminary exploration into the cost burden faced by transgender female patients seeking FFS. Some trends were positive, including a broad range of covered outpatient procedures, from blepharoplasty to midface reconstruction, as well as reduced costs under state-based coverage such as Medicare. However, many procedures were still found to carry high price tags, which are undoubtedly prohibitive for many low-income patients. This study opens up several significant avenues for future research, including a comprehensive analysis across multiple institutions that encompasses both inpatient and outpatient FFS to ensure a representative sample. Furthermore, it is important to examine differences in cost and coverage based on patients’ gender identity, especially given that many outpatient FFS overlap with some of the most commonly performed cosmetic facial surgeries in the United States. Such insights could help clarify whether insurance providers' reluctance to cover these procedures as medically necessary is related to their use in a purely cosmetic context.
